# Development of a rapid field-applicable molecular diagnostic for knockdown resistance (*kdr*) markers in *An. gambiae*

**DOI:** 10.1186/s13071-018-2893-6

**Published:** 2018-05-18

**Authors:** Vera T. Unwin, Shaun Ainsworth, Emily J. Rippon, El Hadji Amadou Niang, Mark J. I. Paine, David Weetman, Emily R. Adams

**Affiliations:** 1 Liverpool School of Tropical Medicine, Pembroke Place, L3 5QA, Liverpool, UK; 2 genedrive plc, Grafton Street, M13 9XX, Manchester, UK; 3 Université Cheikh Anta Diop, 5005 Dakar, Senegal

## Abstract

**Background:**

The spread of insecticide resistance (IR) is a major threat to vector control programmes for mosquito-borne diseases. Early detection of IR using diagnostic markers could help inform these programmes, especially in remote locations where gathering reliable bioassay data is challenging. Most current molecular tests for genetic IR markers are only suitable for use in well-equipped laboratory settings. There is an unmet need for field-applicable diagnostics.

**Methods:**

A single-cartridge test was designed to detect key IR mutations in the major African vector of malaria, *Anopheles gambiae.* Developed on the portable, rapid, point-of-care compatible PCR platform - Genedrive® (genedrive® plc), the test comprises two assays which target single nucleotide polymorphisms (SNPs) in the voltage gated sodium channel (VGSC) gene that exert interactive effects on knockdown resistance (*kdr*): L1014F, L1014S and N1575Y.

**Results:**

Distinct melt peaks were observed for each allele at each locus. Preliminary validation of these assays using a test panel of 70 *An. gambiae* samples showed complete agreement of our assays with the widely-used TaqMan assays, achieving a sensitivity and specificity of 100%.

**Conclusion:**

Here we show the development of an insecticide resistance detection assay for use on the Genedrive® platform that has the potential to be the first field-applicable diagnostic for *kdr*.

## Background

Control programmes for vector-borne diseases, such as malaria, are heavily reliant on the use of insecticides to reduce vector populations. The use of insecticide-treated nets (ITNs), long-lasting insecticidal nets (LLINs) and indoor residual spraying (IRS) plays a major role in the overall reduction in global malaria burden. [1]

Until recently, all ITNs and LLINs were formulated with pyrethroid insecticides [2]. The new development of a chlorfenapyr-based LLN offers a potential alternative, since resistance to pyrethroids is now widespread and has been reported in numerous mosquito species [3].

Knockdown resistance mutations (*kdr*) in the para voltage-gated sodium channel (*Vgsc*) of neurones are one of the principle mechanisms of resistance to pyrethroids and dichlorodiphenyltrichloroethane (DDT) in insects, resulting in an increased tolerance to insecticide exposure, compromising vector mortality [4]. Three mutations in the *Vgsc* gene are linked to pyrethroid and DDT resistance in the principal African vectors of malaria: *Anopheles gambiae*, *An. coluzzii* and *An. arabiensis*. Most common are two mutations to the leucine residue at position 1014 in the wild-type Vgsc-1014 [L1014F (TTA > TTT) and L1014S (TTA > TCA)], located in the hydrophobic segment S6 of domain II (IIS6) resulting in a conformational change preventing access of the insecticide to the active site VGSC protein [5]. The L1014F (‘F’) mutation is the most common *kdr* mutation across diverse insect taxa [6]. L1014S (‘S’) was previously only found in mosquitoes and has recently been detected in the visceral leishmaniasis vector *Phlebotomus argentipes* [7]. Although first detected in West and East Africa, F and S mutations have both been detected across the continent and sometimes co-occur [8]. The mutations do not occur on the same haplotypes, but where both alleles are present, effects on *kdr* are additive [7, 9]. In *An. gambiae* and *An. coluzzii*, a third mutation in the *Vgsc*- Asp1575Tyr (ATA > ATT) has been found in West Africa [10]. The 1575Y (‘Y’) mutation is only present on the F haplotype and acts as an amplifier of resistance to both pyrethroids and DDT [10, 11].

Existing diagnostics for *kdr* markers, i.e. allele-specific PCR (ASPCR), HOLA, SSOP-ELISA, PCR-Dot Blot, FRET/MCA, TaqMan and HRM, were recently compared to a new SimpleProbe® RT-PCR/melt curve assay in *Culex quinquefasciatus*. The melt curve assay was found to be cheaper, faster and more reliable than alternatives [12]. Melt curve assays are designed on the principle that a sequence-specific probe will dissociate away from a DNA duplex at a characterising melting temperature (Tm). In the presence of a mismatched sequence, probe-binding is less efficient, reducing the Tm, and causing a characteristic Tm shift.

Although melt curve-based techniques have advantages over other PCR-based methods, they still require expensive, mains-powered real-time-PCR platforms and skilled technicians [12]. Furthermore, they have only been validated using purified DNA. There is a need for simpler field-applicable molecular tests that could be used in the resource-limited settings where vector control programmes are commonly employed.

Genedrive® is a molecular diagnostics platform that utilises PCR with detection of a fluorescent reporter dye. The total run time of the system ranges from 45 to 90 min depending on assay, and can be utilised direct from the electricity mains or *via* a portable uninterruptable power supply (UPS) which also functions as a battery. While existing Genedrive® tests target pathogens (HCV [13], MTb [14]) and pharmacogenomic mutations (IL-28B [15]) from such diverse clinical samples as plasma, sputum and buccal swabs, respectively, the technology has not been adopted for use with insects.

## Methods

### Primer and probe design

Two separate assays were designed to target three individual SNPs within the *Vgsc* gene, the first to discriminate between the two mutations at the L1014 locus and the other at the 1575Y locus.

Dual-labelled fluorescent, HyBeacon-type molecular probes were designed against the L1014F and 1575Y mutation sequences. The probes were designed to dissociate from the amplicon at around 62 °C, whereas mismatched pairing would yield a reduced Tm shift > 2 °C. The Tm of either probe was predicted *in silico* using OligoAnalyzer 3.1 software (https://www.idtdna.com/calc/analyzer) before empirical determination on the LightCycler480 (Roche Applied Science, Penzberg, Germany) (Table 1). Initial screening experiments were carried out on this higher-throughput platform before transfer to the Genedrive® following optimisation. The NCBI-BLAST software was used to design primers and probes specific to the *An. gambiae* complex. Probes were obtained from ATD Bio (Southampton, UK) and primers from Eurofins (Ebersberg, Germany).

**Table 1:** Primer and probe selections following RT-PCR melt curve screening

Primer/probe	Sequence (5'–3')
1014 Forward primer	TCCCCGACCATGATCTGCCAA
1014 Reverse primer	GCACCTGCAAAACAATGTCATGTAA
1014 Probe	**M**GGAAATTTTGTCG**F**AAGTAA**F**GCAA**P**
1575 Forward primer	AAAGAAAGCTGGTGGATCGC
1575 Reverse primer	TGAAAACACTAACCCTTGGACGA
1575 Probe	**M**TATTATGCAA**F**GAAAAAAA**F**GGGT**P**

F, M and P (in bold) denote a fluorescein labelled deoxythymidine base, a trimethoxystilbene and propanol conjugate, respectively.

### Mosquitoes

The following mosquitoes and DNA samples were used for the initial optimisation of the assays:

(i) *Kdr*-susceptible, (S-form) *An. gambiae* (*s.s.*) (wild-type) and *kdr-*resistant, (M-form) *An. coluzzii* (F/F) mosquitoes (Kisumu and VK7 laboratory reference strains, respectively) were provided by the Liverpool Insecticide Testing Establishment (LITE) at the Liverpool School of Tropical Medicine.

(ii) *Kdr*-resistant (S/S) *An. arabiensis* mosquitoes and dual *kdr*- resistant (FY/FY, FF/FY), pre-extracted *An. arabiensis* mosquito DNA came from recent field collections in Senegal, location and sampling details of which will be provided elsewhere.

(iii) A mixed-population panel of *An. gambiae* (*s.s*.) DNA of known genotypes provided from samples archived at LSTM.

### TaqMan reference standard

Three separate TaqMan assays are routinely used for genotyping F [16], S [16] and Y [10] mutants. Here, we used these tests as reference standards, performed exactly as previously published [10, 16]. Genotypes of all mosquitoes were confirmed using these established TaqMan genotyping assays.

### Lysate preparation

The Genedrive® assay was optimised for use with crude mosquito-leg lysates, although N/Y and Y/Y templates were only available as archived extracted DNA samples. For all the other genotypes, single mosquito legs were incubated in 100 μl nuclease-free water at 95 °C for 20 min.

### DNA extraction and quantification

For comparisons of the limit of detection (LOD) of the assay using purified DNA *versus* crude lysate, total genomic DNA was extracted from L/L and F/F mosquitoes using the Qiagen blood and tissue kit according to the manufacturer’s protocol (Qiagen, Hilden, Germany). Extracted DNA was quantified using the Qubit dsDNA high sensitivity kit according to the manufacturer’s protocol (Thermo Fisher, UK).

### The Genedrive® platform

Details of the Genedrive® platform are published in Duffy et al. [15]. In brief, the device uses a single wavelength optical system (400–470 nm LEDs, 535 nM photodiodes) to read a PCR test cartridge comprised of 3 reaction wells. Additional technical specifications are available at https://www. Genedrive.com/Genedrive-system/documentation.php.

### Genedrive® assay optimisation

Each reaction contained 10 μl of lysate or extracted DNA template, 0.2 μM of the probe, 0.1 μM of forward primer, 4 μM of reverse primer (Table 1) in a total reaction volume of 20 μl of the following: 1 mM MgCl_2_, 0.1 mM dNTPs, 12.5 mM Tris (pH 8.5), KCl 62.5 mM, BSA 0.5 mg/ml, GoTaqMDx 0.075 U/μl and 0.25 μl Excipient (GE Healthcare, Amersham, UK). Several primer/probe sets were screened before the final sets were selected (shown in Table 1). Initial optimisations on the LightCycler480 were carried out under the following cycling conditions: 95 °C for 10 min, followed by 50 cycles of amplification at 95 °C for 10 s, annealing at 62 °C for 10 s, an extension at 72 °C for 10 s, and a final extension at 72 °C for 1 min. This was followed by a melt step increasing the temperature from 42 to 95 °C in 0.5 °C increments with continuous fluorescence acquisition. Genedrive® platform heats and cools rapidly allowing thermocycling for less than 1 s and resulting in a short run time of 50 min. Final reaction conditions used were: 50 cycles of amplification at 95 °C for 0 s (allowed to reach 95 °C only) before reducing the temperature to 62 °C for 10 s, and then raising it to 72 °C for 0 s (again reaching that temperature only momentarily), followed by a final melt step increasing the temperature from 42°C to 80 °C in 0.5 °C increments with continuous fluorescence acquisition, followed by a cooling to 40 °C for 60 s.

### Analytical accuracy

A randomly-chosen panel of 70 *An. gambiae* DNA samples was compiled from previously-collected and TaqMan-genotyped, mosquito samples, and 10 no-template controls were added. The operator of the Genedrive® assay was blinded to the genotypes of the panel.

Sensitivity of the 1014 assay was assessed using: (i) crude lysate and (ii) normalised DNA, from individual mosquitoes. DNA was tested at 1 ng/μl, 100 pg/μl and 10 pg/μl. Crude lysate on the other hand was tested following dilution factors of: 20, 50 and 100, as DNA concentration could not be estimated in crude lysate owing to interference in absorption by liberated proteins. These dilution series were selected based on the amount of DNA a typical extraction might yield from a single mosquito.

### Pooling mosquitoes

Mosquito pools contained a single F/F mosquito with either 3, 5 or 7, L/L mosquitoes. Crude lysates for these pools were obtained as described above following the addition of 100 μl nuclease-free water per mosquito.

### Pooling crude lysates

Lysate pools were constructed by mixing 10 μl of crude lysate from individually lysed mosquitoes in the following F/F to L/L ratios: 1:1, 1:3 and 1:4.

### Pooling DNA

Extracted DNA samples were first diluted in nuclease-free water to a working concentration of 1 ng/μl. ‘DNA pools’ were generated in ratios of 1/4, 1/5, 1/6, 1/7 of F/F:L/L DNA.

## Results

### Primer and probe selection

Candidate primers were screened using WT mosquito lysate as template and the best pair was selected based on peak height fluorescence. Positive control DNA for each genotype was used to screen candidate probes and selection of the final probe was made according to largest observed Tm shifts (°C) between genotypes, in addition to highest peak fluorescence (not shown). Selected primers and probes are shown in Table 1.

### Detection of *kdr* alleles

**Fig. 1:**
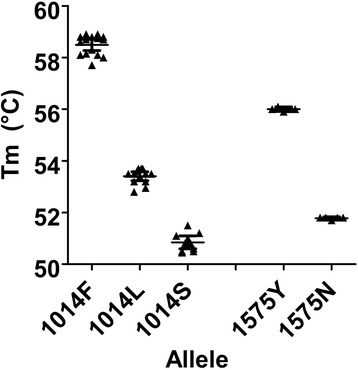
Detection of *kdr* SNPs by melt curve analysis. Alleles are characterised by the melting temperature at which the probe dissociates from its complementary strand located within the target amplicon, producing a drop in fluorescence, which is then automatically converted into a peak by the Genedrive software using the First Order Derivative (FOD). Mean Tm (°C) for each genotype using extracted DNA ± 95% CI; L/L (53.4 ± 0.17), L/F (58.5 ± 0.22), L/S (50.8 ± 0.25), N/N (51.8 ± 0.06) and N/Y (56 ± 0.09)

**Fig. 2:**
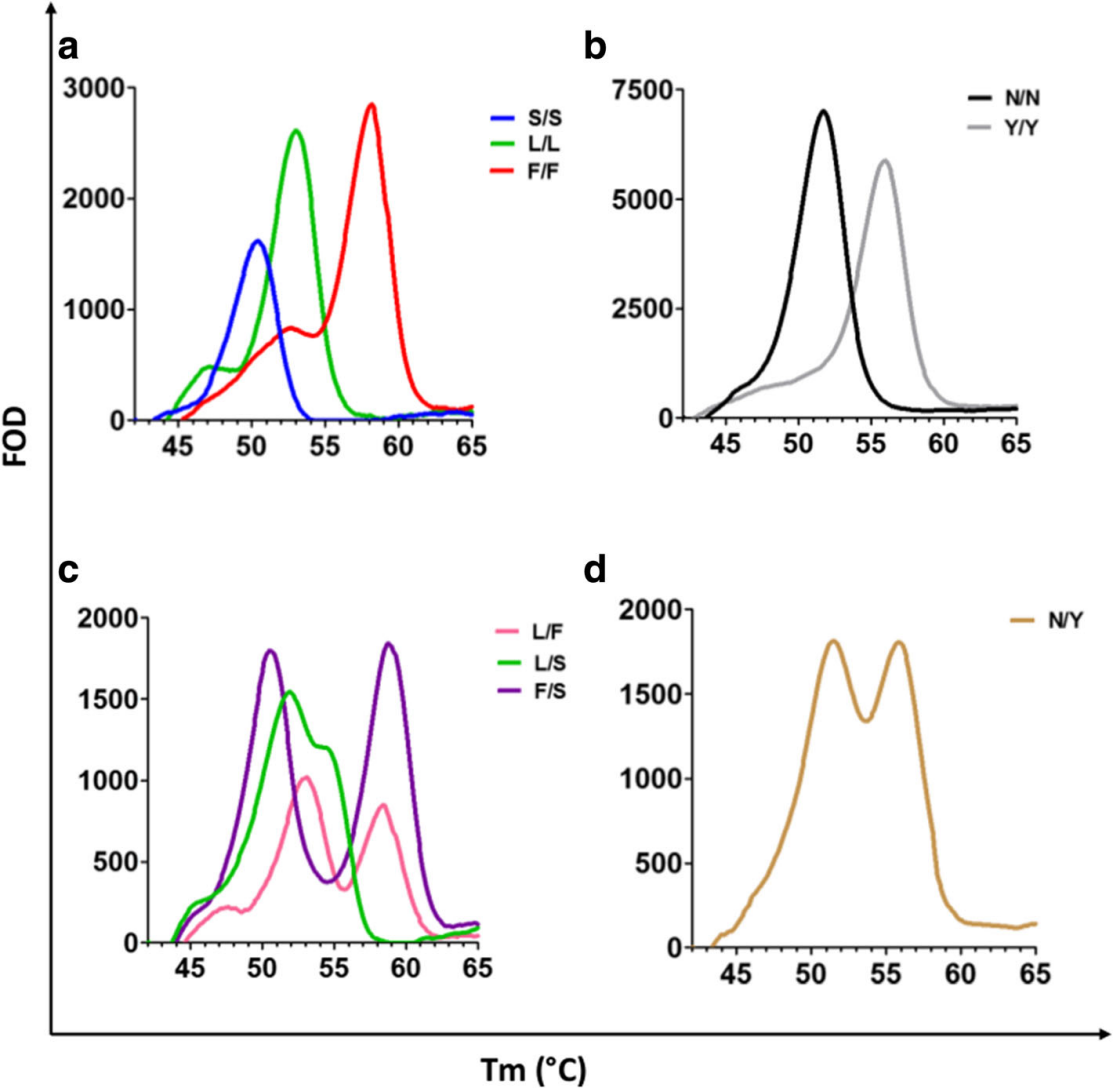
FOD melt curves of *kdr* genotypes. FOD melt curves of homozygous genotypes at the 1014 locus (**a**): S/S (blue), F/F (red) and L/L (WT, green) and at the 1575 locus (**b**): N/N (WT, black) and N/Y (grey). Heterozygous genotypes are shown in **c** at the 1014 locus: L/F (pink), L/S (green) and F/S (purple), and **d** at the 1575 locus: N/Y (brown)

Using the Genedrive® platform, all genotypes were determined based on the presence of Tm specific peaks. Discernible melt peaks were observed for all alleles in both the 1014 or 1575 assays as highlighted in Fig. 1. In each case the peaks between wild-type and mutant were separated by Tm shifts of at least 2 °C. (Fig. 2a-d) In comparison to extracted DNA, average Tm peaks of L and F alleles were slightly higher in lysates (Table 2).

**Table 2:** Comparison of melting temperature of lysate and extracted DNA template

	Tm range (°C)		Average Tm ± SD	
Allele	DNA extract (*n*)	Crude lysate (*n*)	DNA extract	Crude lysate
1014F	57.7–58.9 (15)	59–59.55 (12)	58.49 ± 0.39	59.31 ± 0.14
1014L	52.8–53.7 (13)	53.9–54.7 (11)	53.4 ± 0.29	54.29 ± 0.21

### Limits of detection

**Fig. 3:**
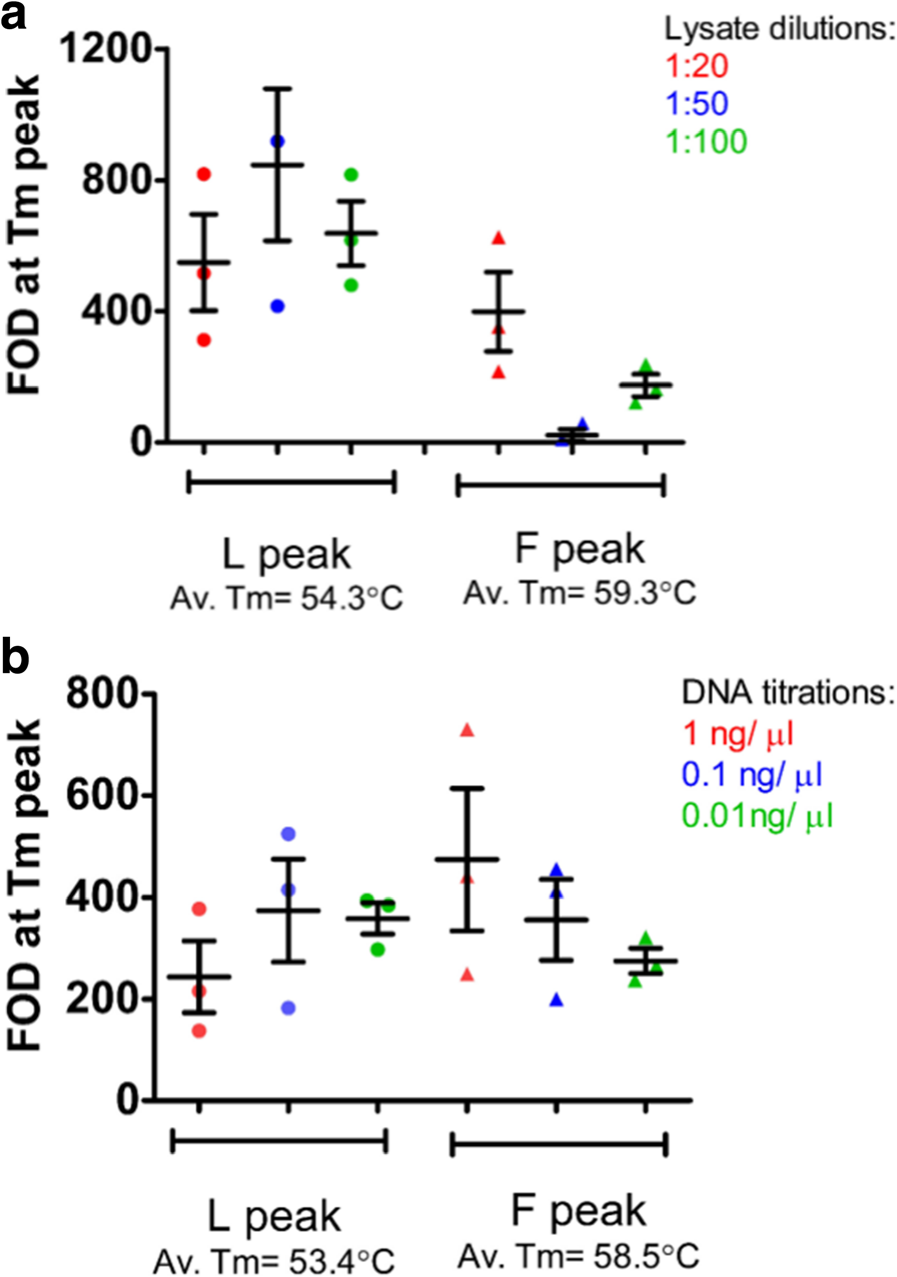
Titration of crude lysates and extracted DNA to assess functional sensitivity of the 1014 assay. Different titrations of crude lysates (**a**) and DNA extracts (**b**) of F/F and L/L mosquitoes were mixed at a 1:1 ratio. Each dot represents a single peak (one biological replicate) and bars represent SEM

The sensitivity of the 1014 assay when using individual mosquitoes was tested using: (i) extracted DNA at 1 ng/μl, 100 pg/μl and 10 pg/μl and (ii) crude lysate at dilutions 1/20, 1/50 and 1/100. Figure 3 shows distinct Tm peaks (Tm shift between alleles ≥ 2 °C with FOD ≥ 100) detected across all dilutions using DNA template; however, the F-peak starts to drop when using lysates. This suggests pooling of lysates is less sensitive than pooling DNA.

Sensitivity and specificity was determined by screening a panel of *An. gambiae* (*s.s.*) DNA samples of known genotype identified using TaqMan assays as a reference standard. Table 3 shows that both 1014 and 1575 tests had 100% sensitivity and specificity.

**Table 3:** Sensitivity and specificity of 1014 and 1575 Genedrive® assays compared to those determined using TaqMan in a test panel of 70 *An. gambiae* DNA samples

			TaqMan								
			1014 loci						1575 loci		
			LL	LF	FF	LS	SS	FS	NN	NY	YY
Genedrive®	1014 loci	LL	1	–	–	–	–	–	–	–	–
		LF	–	3	–	–	–	–	–	–	–
		FF	–	–	58	–	–	–	–	–	–
		LS	–	–	–	–	–	–	–	–	–
		SS	–	–	–	–	1	–	–	–	–
		FS	–	–	–	–	–	9	–	–	–
	1575 loci	NN	–	–	–	–	–	–	52	–	–
		NY	–	–	–	–	–	–	–	14	–
		YY	–	–	–	–	–	–	–	–	4

### Detection of genotypes in mosquito pools

To increase throughput, the sensitivity of the 1014 assay using pools of mosquitoes was investigated. These included: (i) pooling mosquitoes for lysate preparation and (ii) pooling lysates obtained from individual mosquitoes. Mosquito pools contained a single homozygous mutant F/F mosquito with either 3, 5 or 7 homozygous WT L/L mosquitoes. Lysates for these pools were generated as described earlier followed by the addition of 100 μl nuclease-free water per mosquito.

**Fig. 4:**
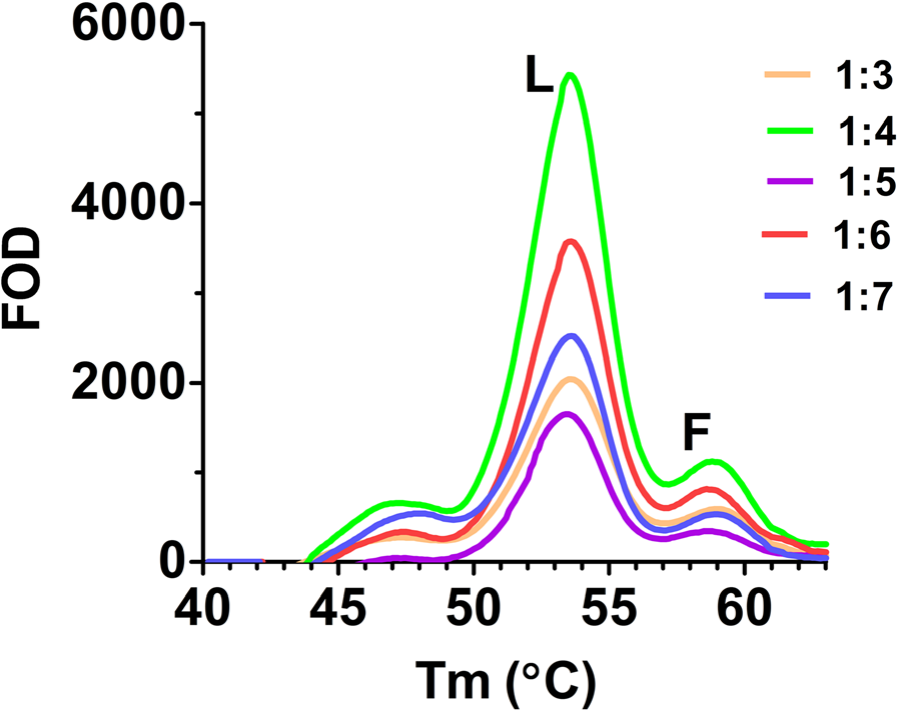
Pooled DNA from 1014F and WT mosquitoes. DNA was extracted from F/F and L/L mosquitoes and pooled in the following F/F:L/L ratios: 1:3 (orange), 1:4 (green), 1:5 (purple), 1:6 (red) and 1:7 (blue). Each line represents the melt curve of a single replicate

Pooling lysates from individual mosquitoes resulted in poor detection of the F allele when diluted in a background of L alleles; a theoretical pool of 2 mosquitos (a single L/F heterozygote and an L/L homozygote) failed to produce a discernible peak (data not shown). Using extracted and normalised DNA significantly improved pooling results. Using 1 ng/μl of DNA, ratios of 1/4, 1/5, 1/6, 1/7 of homozygous F/F: L/L DNA were tested. Figure 4 shows example melt curves of these pools. Results show two discernible peaks at 58.8 and 53.6 °C, corresponding to the F and L peaks, are observed at a 1/7 ratio (equivalent to a pool of 4 mosquitoes: one F/F and three L/L mosquitoes). Since the Genedrive® cartridge contains 3 wells, there is potential for 12 DNA samples to be screened in one run.

## Discussion

Here we have developed a method using the Genedrive® platform for the detection of three mutations that indicate insecticide resistance at the 1014 and 1575 loci in *An. gambiae.* Genedrive® uses end-point melt analysis to give rapid results with limited sample pre-processing, [14, 15] and is shown to be highly accurate when tested on a panel of 80 samples. Although wet reagents were used throughout this method development work, like all other commercially-available Genedrive® tests (HCV [13], MTb [14] and IL-28B [15]), the final optimised formulation will be lyophilised within the cartridges so that only the addition of template is required, and also eliminating the need for any cold chain storage. Additionally, the Genedrive® platform is very easy to use with only a single button required for its operation thereby simplifying the workflow and reducing the need for skilled operators. The Genedrive® platform is portable weighing less than 600 grams [17] and could be used directly in the field for ‘point-of-care’ monitoring or in decentralised, minimally equipped laboratories.

Several novel diagnostics have been developed for detection of *kdr* in mosquitoes in attempts to simplify assays and reduce costs, yet TaqMan assays, which use expensive fluorescent probes, remain the most commonly used assay [12]. Melt-curve assays have been previously developed to simultaneously detect both F and S alleles, which reduces labour and the reagent costs whilst producing easily interpretable results [18]. However, these methods still require substantial upfront costs for equipment and require skilled expertise to perform.

The 1014 assay has been designed so that only a single probe is required to simultaneously discriminate between the L, F and S alleles, whereas the 1575 assay requires a second probe for the detection of the N and Y alleles, each using their assay-specific primer pairs.

By comparison, detection of these mutations by conventional TaqMan methods would require 5 different probes: Y, F and S- mutant probes, as well as WT probes for each 1014 and 1575 sequence. The costs of reagents are thus significantly reduced. In addition to the simple workflow and reduced costs on reagents, the cost of the GeneDrive machine itself is estimated to be less than a third of the price of a 48 well rt-PCR machine (US $19,000–20,000 [16]).

It should be noted that we observed an increase in peak Tm across 1014F and 1014L genotypes when comparing sample lysates with purified DNA (Table 3). This is most likely due to the differing background of salt concentrations in the lysates [19]. For automated Genedrive genotype detection, a defined Tm range would require determination through further testing on different lysates to account for intra-individual variability. Although a larger sample size to determine this would result in a broader Tm range, it is important to note that the Tm shift between each of the alleles remains relatively constant. This could be accounted for in the algorithm for automation of the readout.

The Genedrive cartridge designed here allows for several IR markers to be detected using only two of the three available channels in a cartridge. This allows potential to incorporate a third assay for additional markers or, alternatively the assays could be separated into different cartridges to process multiple samples in one run.

Using pooling strategies, our results show that there is potential to screen DNA from up to 12 mosquitoes for 1014F mutations in a single run (50 min). There is also potential to upscale analysis by increasing numbers of cartridges or machines. Pooling samples for GeneDrive analysis could allow either qualitative detection of the presence of resistance alleles in a population of mosquitoes or quantitative detection of spatial or temporal variation. Pooling samples has the general limitation of making interactions between alleles within loci (dominance effects) and across loci (epistasis) difficult to detect. For the markers considered here, 1014F and 1014S appear to no more than partially recessive [20, 21] and 1575Y occurs only on a 1014F haplotype [10]. Therefore, useful information can be gained from pooled data, and in terms of vector control management, if a frequency threshold is determined, detection of variation in allele frequencies at resistance phenotype-informative markers from pooled mosquitoes would warrant examination of the insecticide used in a locality. However, for extension to additional markers the importance of dominance level and epistasis must be considered when adopting a pooling strategy.

These assays could be expanded to include different target site mutations and other disease vectors, such as sand flies [22] or triatomine bugs [23], where *kdr* mutations have also been reported. However, although melt analysis-based assays are useful for the detection of DNA substitutions or indels, it is more difficult to detect multiplication mutations, e.g. duplication of detoxifying enzymes, since melt curve analysis is only semi-quantitative.

## Conclusions

This study describes the development and validation of two simple molecular assays for *kdr* genotyping in *An. gambiae* mosquitoes. Our results show accurate detection of the L1014F, L1014S and N1575Y *kdr*-associated SNPs in *An. gambiae*. Development on the Genedrive® platform presents a viable methodology for applying these assays as a field-applicable diagnostic in low-resource settings.

## Abbreviations

DDT: Dichlorodiphenyltrichloroethane
IRS: Indoor residual spraying
ITN: Insecticide-treated nets
*kdr*: Knockdown resistance
LLIN: Long-lasting insecticide treated nets
RT-PCR: Real-time polymerase chain reaction
SNP: Single nucleotide polymorphism
Tm: Melting temperature
VGSC: Voltage gated sodium channel
